# Flaxseed Supplementation in Chicken Feed Accelerates *Salmonella enterica* subsp. *enterica* Serovar Enteritidis Clearance, Modulates Cecum Microbiota, and Influences Ovarian Gene Expression in Laying Hens

**DOI:** 10.3390/biom13091353

**Published:** 2023-09-06

**Authors:** De Wang, Boheng Ma, Ziwei Liao, Wenjing Li, Tiejun Zhang, Changwei Lei, Hongning Wang

**Affiliations:** 1 College of Life Sciences, Sichuan University, Chengdu 610044, China; wangde@stu.scu.edu.cn (D.W.); maboheng@stu.scu.edu.cn (B.M.); 2021222040034@stu.scu.edu.cn (Z.L.); 2021222040029@stu.scu.edu.cn (W.L.); zhangtiejun@stu.scu.edu.cn (T.Z.); 2 Key Laboratory of Bio-Resource and Eco-Environment of the Ministry of Education, Chengdu 610064, China; 3 Animal Disease Prevention and Food Safety Key Laboratory of Sichuan Province, Chengdu 610064, China

**Keywords:** *S.* Enteritidis, chicken, flaxseed, gut microbiota, transcriptome, ovarian health

## Abstract

*Salmonella* is a foodborne pathogen that poses a serious threat to both human and animal health and food safety. Flaxseed is rich in unsaturated fatty acids; has anti-metabolic syndrome, anti-inflammatory, and neuroprotective properties; and may be a potential source of feed additives. To investigate the impact of flaxseed on *Salmonella*-infected laying hens, we administered *Salmonella enterica* subsp. *enterica* serovar Enteritidis (*S.* Enteritidis) after adding flaxseed to the feed of laying hens (15% [750 mg/kg]). *S.* Enteritidis colonization was reduced and its clearance was accelerated from the laying hens. Furthermore, flaxseed supplementation mitigated the damage to the ileum caused by *S.* Enteritidis. We analyzed alterations in intestinal flora through 16S rRNA amplicon sequencing. *S.* Enteritidis infection increased the abundance of *Akkermansia* and triggered the host inflammatory response. Conversely, the addition of flaxseed to the feed increased the abundance of beneficial intestinal bacteria, such as *Lactobacilli* and *Bacteroides*. Ovarian health is important for egg production performance in laying hens and our findings indicate that *S.* Enteritidis can persist in the ovaries for an extended period. Therefore, we further performed transcriptome sequencing analysis of ovarian tissues on day seven after *S.* Enteritidis infection. *S.* Enteritidis infection leads to altered ovarian gene expression, including the downregulation of lipid metabolism and growth and development genes and the upregulation of host immune response genes in laying hens. The upregulation of genes associated with growth and development may have stimulated ovarian growth and development.

## 1. Introduction

Foodborne diseases have high morbidity and mortality rates worldwide and substantially threaten human and animal health. *Salmonella* is one of the most widely transmitted foodborne pathogens in poultry and humans and is a major cause of foodborne outbreaks and infections worldwide [1,2]. Approximately 93 million intestinal infections and 155,000 deaths are attributed to *Salmonella* annually [3]. There are more than 2600 known *Salmonella* serovars with different pathogenicities and host specificities. *Salmonella enterica* subsp. *enterica* serovar Enteritidis (*S.* Enteritidis) is one of the most common pathogens isolated from chickens. It causes various intestinal diseases, such as inflammation, barrier dysfunction, and the disruption of the intestinal microbiota [4,5]. *S.* Enteritidis is the most common cause of foodborne infections in China (26.82%) [6] and is a major serotype found in chickens in Central China [7].

The gut microbiota can influence host energy homeostasis, metabolism, immunity, and the endocrine system and it plays a key role in maternal metabolism and offspring growth [8]. Ecological dysregulation of the gut microbiota may be a potential causative factor in the development of ovarian dysfunction and reduced fertility [9]. Vertical transmission is an important route of *Salmonella* transmission in poultry production and can lead to significant reductions in laying performance and egg quality [10]. *Salmonella* primarily colonizes the cecum of chickens, invades the intestinal epithelial and dendritic cells, and reaches the submucosal layer before it is phagocytosed by macrophages [11]. In poultry, the gastrointestinal and reproductive tracts are connected to the cloacae. Therefore, gastrointestinal bacteria may be transferred to the reproductive tract during excretion, affecting ovarian health [12]. Previous studies have shown that *S.* Enteritidis not only colonizes reproductive tissues but is also present in the ovary at a higher rate than any other reproductive tissue [13,14].

Flaxseed is considered a functional food with potential health benefits. It is rich in fatty acids required for the normal physiological functioning of organisms [15]. Flaxseed is a rich terrestrial source of polyunsaturated fatty acids (PUFA), especially alpha-linolenic acid (ALA), and partially defatted flaxseed meal contains more than 33% ALA in dry matter [16]. Therefore, flaxseed is incorporated into chicken diets as an ALA precursor [17]. ALA has anti-metabolic syndrome, anti-inflammatory, antioxidant, anti-obesity, and neuroprotective properties [18]. Dietary flaxseeds have the potential to enhance innate immunity against *Salmonella* colonization in chickens and flaxseed mucilage has antibacterial activity against *Salmonella* [19,20].

Flaxseed can alter estrogen levels, inflammation status, epigenetics, angiogenesis, and apoptosis microenvironments in ovarian tumors of laying hens and increase the lifespan of chickens [21]. However, studies are still lacking on the effects of adding flaxseed to feed on *Salmonella* infection in laying hens. Therefore, we investigated the effect of adding flaxseed to the feed of laying hens on *Salmonella* colonization. We also evaluated changes in ovarian gene expression in chickens infected with *Salmonella* and fed with flaxseed.

## 2. Materials and Methods

### 2.1. Bacterial Strains

*S.* Enteritidis ATCC 13076 (stored in the Animal Disease Prevention and Food Safety Key Laboratory of Sichuan Province, Chengdu, China) was incubated for 16 h at 37 °C in lysogenic broth. The bacterial suspension was spread onto XLT4 agar plates (Beijing Landbridge Technology Limited by Share Ltd., Beijing, China) and incubated at 37 °C for 24 h before counting.

### 2.2. Animal Experiments

This study was approved by the Ethics Committee of College of Life Sciences, Sichuan University (protocol code: SCU220530001 and date of approval 30 May 2022). In total, 100 120-day-old laying hens were obtained from a commercial egg farm (Sichuan Sundaily Farm Eco-Food Co., Ltd., Mianyang, China). The hens were randomly divided into four subgroups (n = 25 in each group), housed in separate enclosures, and allowed free access to food and water.

Hens in the AS and A groups were continuously fed flaxseed-containing [15% (750 mg/kg)] feed for days 1–28 [22,23]. The S and NC groups were fed a regular diet. The AS and S groups were then orally administered 1 × 10^8^ colony-forming units (CFU) of *S.* Enteritidis daily from days 29 to 31. Groups A and NC were fed equal amounts of lysogeny broth medium. Five chickens from each group were randomly selected for euthanasia using 5% pentobarbital solution (50 mg/kg body weight) on days 3, 7, 14, 21, and 28 after *S.* Enteritidis infection. Samples of cecum, heart, spleen, liver, ovary, and ileum were collected under sterile conditions. Ileum samples were preserved in 10% paraformaldehyde. Other tissue samples were stored at −80 °C pending use in subsequent experiments.

### 2.3. DNA and RNA Extraction and RNA Reverse Transcription

DNA was extracted from the collected cecum contents using a TIANamp Stool DNA Kit (Tiangen, Beijing, China) according to the manufacturer’s instructions. The concentration and quality of the extracted DNA were determined using a NanoDrop spectrophotometer (Thermo, Beijing, China) and agarose gel electrophoresis. The extracted DNA was stored at −20 °C until further analysis.

At 7 dpi, ovarian samples were collected from five chickens in each group. Ovarian RNA was extracted using an RNeasy Kit (QIAGEN, Hilden, Germany) according to the manufacturer’s instructions. The extracted RNA was stored in a refrigerator at −80 °C before further analysis and then used for transcriptome sequencing analysis and reverse transcription-polymerase chain reaction (RT-PCR). A HiScript II 1st Strand cDNA Synthesis Kit (Vazyme, Nanjing, China) was used to perform RT-PCR on the extracted RNA according to the manufacturer’s instructions. The RT-PCR cycling conditions were as follows: 25 °C for 5 min, 50 °C for 15 min, and 85 °C for 2 min. The cDNA obtained was stored at −80 °C for use in subsequent experiments.

### 2.4. Detection of S. Enteritidis Abundance via Plate Counting

At 3, 7, 14, 21, and 28 dpi, five chickens in each group were randomly selected for euthanasia and 50 mg samples of the cecum, heart, spleen, liver, and ovaries were collected. First, 10 mL of PBS was added to the samples and shaken well. Next, 1 mL of the bacterial solution was homogenized in 9 mL of PBS and the serially diluted samples were coated onto XLT4 agar plates. Finally, *S.* Enteritidis colonies were counted after incubation at 37 °C for 24 h under aerobic conditions. The results are expressed as log_10_ CFU/g. All analyses were repeated thrice.

### 2.5. Histopathological Examination of the Ileum

The ileal tissues were preserved in 10% neutral formaldehyde fixative for 24 h. The fixed tissues were then subjected to automatic dehydration, paraffin embedding, sectioning (5 µm), and hematoxylin–eosin (HE) staining. After dehydration and sealing, the sections were imaged using a digital microscope and each section was observed at 40× magnification. If a general lesion was observed, the area was selected to acquire 100× or 400× images for further examination. Tissue section pathology scoring was performed to determine the extent of the lesion, as previously described [24].

### 2.6. 16S rRNA Amplicon Sequencing and Bioinformatics Analysis

PCR amplification was performed on the V3–V4 hypervariable region of the bacterial 16S rRNA gene using the isolated cecum genomic DNA as a template. The primers used were 338F (5′-ACTCCTACGGGAGGCAGCAG-3′) and 806R (5′-GGACTACHVGGGTWTCTAAT-3′). AMPure XP magnetic beads were used to purify the PCR amplification products. After the libraries were labeled, an Agilent 2100 Bioanalyzer (Agilent Technologies, Santa Clara, CA, USA) was used to check the fragment range and concentration of the library. Qualified libraries were sequenced on a HiSeq platform (HiSeq 2500, Illumina, San Diego, CA, USA) according to the insert size.

Raw data were filtered and joint sequences were removed using Cutadapt (v 1.18) [25]. PEAR software (v 0.9.8) was used to stitch pairs of reads into a sequence based on the overlapping relationship between paired-end reads [26]. Sample data were segmented from the spliced data according to each sample barcode sequence and primer sequence and sequence orientation correction was performed. Bases with quality values below 20 in the tail of the reads were excised using PRINSEQ (v 0.20.4). N-containing and short sequences were filtered after quality control and low-complexity sequences were filtered out [27]. Operational taxonomic unit clustering analysis was performed using Usearch software (v 11.0.667) [28]. Alpha diversity was analyzed using MOTHUR (v 1.43.0) [29]. Using the R package mixOmics (v 6.10.6), partial least squares-discriminant analysis (PLS-DA) models were constructed based on species abundance matrices and sample grouping data. To evaluate the fitting effectiveness of the PLS-DA model, two metrics were used: R2 and Q2. R2 indicates the rate of explanation of the X and Y matrices of the constructed model and Q2 labels the predictive ability of the model. Theoretically, the closer the values of R2 and Q2 are to 1, the better the model is, and the lower the value is, the more inaccurate the model’s fitting accuracy is. The bar charts at different classification levels were drawn using the R package (v 3.6.0) and R package gplots (v 3.0.1.1). LEfSe clustering and LDA were performed using LEfSe software (v 1.1.0) [30].

### 2.7. RNA Sequence Library Construction and Sequencing

RNA sequence libraries were prepared using the standard Illumina Novogene protocol. mRNA was enriched from total RNA using poly-A oligonucleotide-linked magnetic beads with integrity values greater than 8.0. Double-stranded cDNA was synthesized using random hexamer primers and purified using AMPure XP beads. Concentrate inserts of the expected size were selected and transcriptome analysis was performed.

Data were evaluated and the quality was controlled. Quality assessment of the raw sequencing data was performed using FastQC (v 0.11.2) and quality clipping was performed using Trimmomatic (v 0.36) to obtain relatively accurate and valid data [31]. RNA-seq was performed for evaluation. HISAT2 (v 2.1.0) was used to match valid sample data to the reference genome and analyze the mapping information [32]. RSeQC (v 2.6.1) was used to perform redundant sequence analysis and insertion fragment distribution based on the comparison results [33]. Homogeneous and genome structure distribution analysis was then performed based on the comparison results using Qualimap (v 2.2.1) [34]. Statistical analysis of gene coverage and chromosome sequence distribution was performed using BED Tools (v 2.26.0) [35]. Expression difference analysis was also performed. Differential gene expression analysis was conducted using DESeq2 (v 1.12.4) and differential expression was visualized as previously described [36]. Venn diagrams were drawn based on the results of the variance analysis and clustering analysis was performed. Finally, a gene enrichment analysis was performed. GO enrichment analysis was performed using top GO [37]. KEGG pathway enrichment analysis were performed using a cluster profiler (v 3.0.5) [38]. An association analysis network diagram was drawn based on gene function enrichment analysis [30].

### 2.8. Quantitative RT-PCR Validation

The *rpl13*, *calr*, *cd24*, *tagln*, and *rps27a* genes from the ovary were selected to validate the accuracy of the gene expression data obtained via RNA sequencing. Primers were designed and synthesized by Sangon Biotech (Shanghai, China), the sequences of which are listed in Table 1. The cDNA samples from the ovaries were subjected to RT-qPCR using SsoFast EvaGreen Supermix (Bio-Rad Inc., Hercules, CA, USA) according to the manufacturer’s instructions on a Bio-Rad real-time PCR system (CFX Maestro 1.1 or 3.0; Bio-Rad Inc.). The reaction protocol for all genes was as follows: 5 min at 95 °C followed by 40 cycles of 10 s at 95 °C and 30 s at 60 °C.

### 2.9. Statistical Analysis

S. Enteritidis abundance was converted to log_10_ CFU per gram. The results of the *S.* Enteritidis abundance, ileal pathology score, and transcriptome gene expression abundance validation were analyzed by one-way ANOVA using SPSS software (version 20.0; IBM, Armonk, NY, USA). Statistical assessment of the changes in microbial abundance of cecum contents was conducted by a Wilcoxon rank sum test.

## 3. Results

### 3.1. Flaxseed Reduces S. Enteritidis Colonisation in Chickens

**Figure 1:**
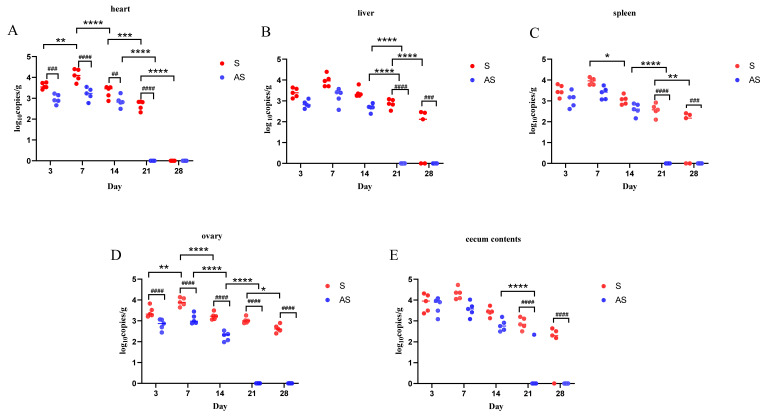
*S.* Enteritidis abundance in the heart (**A**), liver (**B**), spleen (**C**), ovary (**D**), and cecum contents (**E**). Data are expressed as the mean ± standard deviation. *, *p* < 0.05; **, *p* < 0.01; ***, *p* < 0.001; ****, *p* < 0.0001; #, *p* < 0.05; ##, *p* < 0.01; ###, *p* < 0.001; ####, *p* < 0.0001.

We evaluated the abundance of *S.* Enteritidis in the laying hens. Chickens were fed in the specific pathogen free (SPF) environment and all chickens tested negative for *Salmonella* until experimentally infected with *S.* Enteritidis. We evaluated the level of *Salmonella* colonization in the untreated group (NC) and the group with flaxseed added to the feed (A) which had negative *Salmonella* cultures throughout the study. At three days post-infection (dpi), S. Enteritidis was found in all tissues of the S group (*S.* Enteritidis infection) and the AS group (flaxseed added to feed + *S.* Enteritidis infection). In addition, the abundance of *S.* Enteritidis in group S was higher than that in group AS (*p* < 0.05) (Figure 1). The abundance of *S*. Enteritidis in the ovary was 3.827 CFU/g in group S and 2.698 CFU/g in group AS (Figure 1D). At seven dpi, the highest abundance of *S.* Enteritidis was found in all tissues of the S and AS groups. The number of *S*. Enteritidis in the cecum contents was as high as 4.728 CFU/g (Figure 1E). At 14 dpi, the number of *S.* Enteritidis gradually decreased. Particularly, the abundance of *S.* Enteritidis in the cecum, spleen, ovary, and heart was lower than at seven dpi (*p* < 0.05) (Figure 1A,C–E). At 21 dpi, *S.* Enteritidis was scarcely isolated from the tissues of the AS group whereas the *S.* Enteritidis count remained high in the S group samples. Compared with that at 14 dpi, there was no marked decrease in the abundance of *S.* Enteritidis in any tissue of the S group except for the heart (*p* < 0.05) (Figure 1B–E). At 28 dpi, *S.* Enteritidis persisted in the ovarian tissues of the S group (2.718 log_10_ CFU/g) (Figure 1D).

### 3.2. Flaxseed Attenuates Barrier Damage to the Ileum Caused by S. Enteritidis

**Figure 2:**
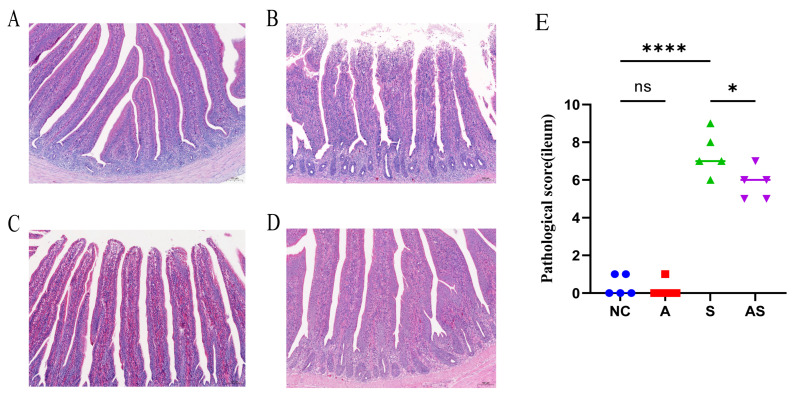
Hematoxylin and eosin staining for the histopathological analysis of intestinal tissues in the NC (**A**), S (**B**), AS (**C**), and A (**D**) groups. Scale bars, 100 µm. Ileal pathology was scored in H- and E-stained ileal tissue sections (**E**). Blue for NC group, red for A group, green for S group, purple for AS group. ns, not different; ****, *p* < 0.0001; *, *p* < 0.05 (by a Mann–Whitney U test).

As the addition of flaxseed to the feed reduced *Salmonella* abundance in laying hens, it is possible that flaxseed plays a role in protecting the intestinal barrier of laying hens, attenuating the greater translocation of *Salmonella* to tissues and organs. Therefore, we investigated the effect of flaxseed on intestinal morphological changes caused by *S.* Enteritidis. Hematoxylin and eosin (H and E) staining showed that groups NC and A had intact ileocecal villi that were structurally complete and tightly arranged (Figure 2A,D). In group S, the mucosal epithelial cells of the ileum were detached and the mucosal lamina propria was degenerated and necrotic to varying degrees; the necrotic areas were accompanied by inflammatory cell infiltration (Figure 2B). In the AS group, the intestinal mucosal layer, submucosal layer, muscularis propria, and outer membrane of the ileum were more structurally intact, with more cellular components in the mesenchyme around the small intestinal glands and a small amount of inflammatory cell infiltration in the mesenchyme (Figure 2C). Pathological scores showed NC and S groups, AS and S groups are marked. The score of group S was higher than that of group NC (*p* < 0.0001) and that of group AS was lower than that of group S (*p* < 0.05) (Figure 2E). Addition of flaxseed to the feed reduced the score of ileal damage.

### 3.3. Effect of Flaxseed on the Microbial Composition of the Cecum Contents of Laying Hens

The 16S rRNA gene from the cecal microbiome at five different sampling time points was sequenced and multiple metrics were used to assess the α-diversity of the samples. At tree dpi, the α-diversity was lower in the AS group than in the S group (*p* < 0.05). Moreover, at 7 and 14 dpi, the α-diversity was lower in group A than in group NC (*p* < 0.05). Finally, at 21 dpi, the α-diversity of the AS group was slightly higher than that of the S group; however, the difference was not statistically (*p* > 0.05). At 28 dpi, the α-diversity of the S group was higher than that of the NC group (*p* < 0.05). There was no marked difference in α-diversity between the AS and S groups but both were higher than that in the NC group. There was no notably change in α-diversity of the NC and A groups at all five observed time points. The α-diversity of the S group first decreased and then increased and only at 28 dpi was the colony abundance higher than that of the NC group. In contrast, the α-diversity of the AS group continued to increase, with the highest α-diversity at 28 dpi, considerably higher from that at 3 dpi (*p* < 0.05) (Figure S1).

**Figure 3:**
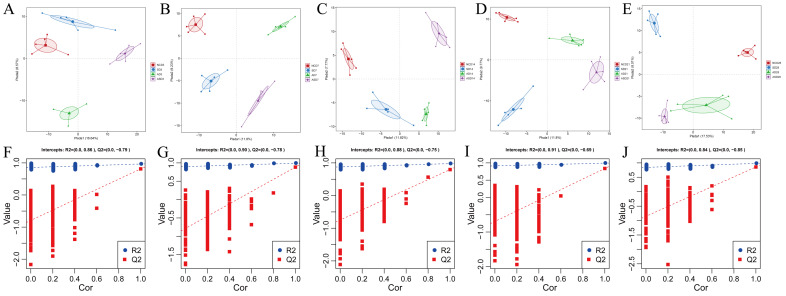
PLS-DA plot of the microbiome of the cecum contents at 3 (**A**), 7 (**B**), 14 (**C**), 21 (**D**), and 28 (**E**) dpi. Validation plots of PLS-DA data for these five groups [(**F**) (3 dpi), (**G**) (7 dip), (**H**) (14 dpi), (**I**) (21 dpi), and (**J**) (28 dpi)].

The R2 and Q2 values of the partial least squares-discriminant analysis (PLS-DA) are both greater than 0.5 and close to 1, indicating a good fit of the PLS-DA model (Figure 3F–J). The results showed that S. Enteritidis and linseed could affect the microbial community structure of the cecum contents of laying hens in the long term (Figure 3A–E). Meanwhile, the microbial community changes with the growth of laying hens (Figure 3A–E).

**Figure 4:**
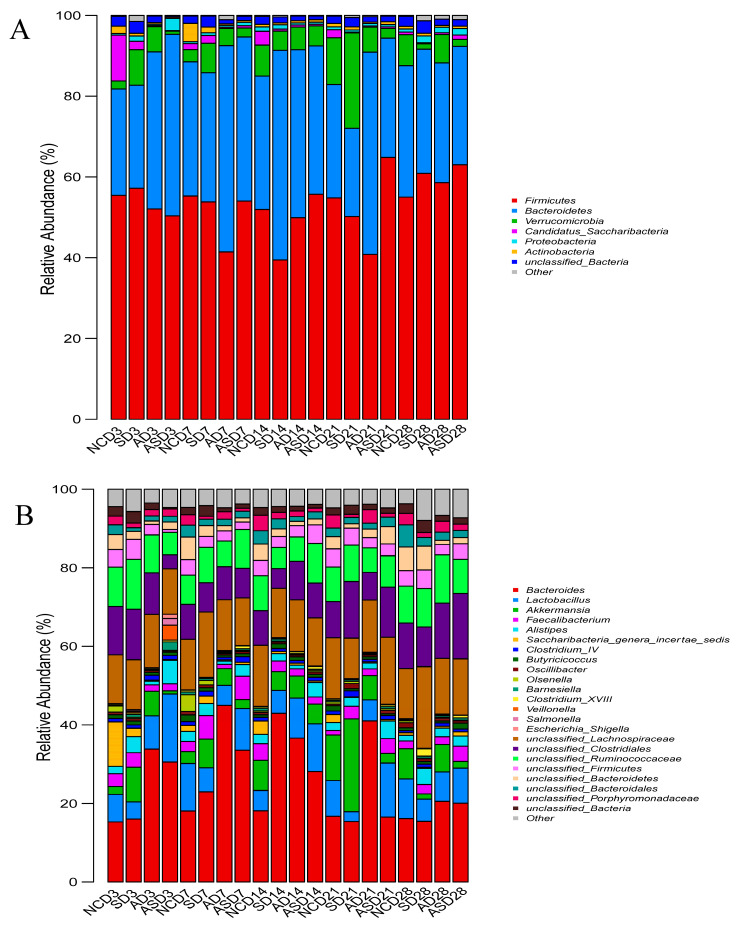
Mean relative abundance of cecal microbiota at the phylum (**A**) and genus (**B**) levels. NC, S, A, and AS represent the different groups and the value after the group label indicates the dpi.

The relative abundances of the cecal microbiota at the phylum and genus levels were compared. Analyses of changes in colony abundance were performed using the Wilcoxon rank sum test (Table S1). Yet, the results of the analysis of variance showed no marked change in colony abundance (*p* > 0.05). However, through the relative abundance of flora, we can still observe the effect of *Salmonella* and flaxseed on the microbial community composition of the cecum of laying hens. At the phylum level, Firmicutes and Bacteroidetes were the dominant microbial groups. In the AS group, the abundance of Firmicutes increased over time whereas that of Bacteroidetes continuously decreased. The abundance of Firmicutes in the S group first decreased and then increased whereas the abundance of Bacteroidetes showed the opposite trend. The relative abundance of Verrucomicrobia was highest in the S group at 3, 7, and 21 dpi (Figure 4A).

At the genus level, *Bacteroides*, *Lactobacillus*, and *Akkermansia* were predominant. The abundance of Bacteroides in the AS and S groups showed a trend of increasing and then decreasing. Except, at 21 dpi the abundance of *Bacteroides* and *Lactobacillus* was higher in the AS group than in the S group. At 3, 7, and 21 dpi, the abundance of *Akkermansia* was higher in the S group than in the AS group (Figure 4B).

**Figure 5:**
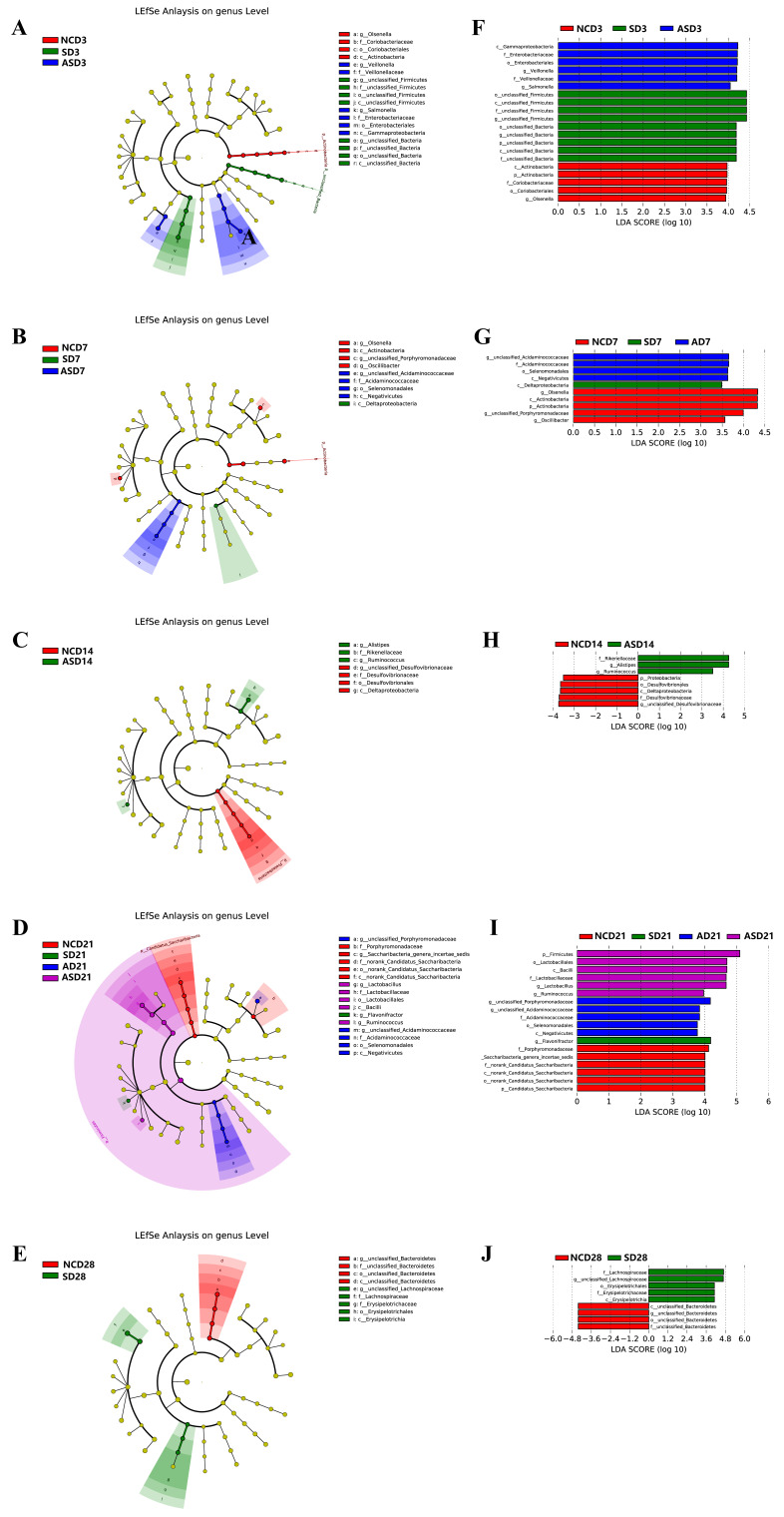
LEfSe analysis of the microbial community in the cecum at five sampling time points. The LEfSe plots showed differences between the four groups of microbial strains (**A**–**E**). The different groups are represented by different colors, the microbiota that plays an important role in the different groups is represented by nodes of corresponding colors, and the organism markers are indicated by colored circles. The microbiota that does not play a role in the different groups is indicated by yellow nodes. From inside to outside: phylum, order, family, and genus. Linear discriminant analysis (LDA) plots (**F**–**J**). Different colors represent the microbial taxa that play a substantial role in different groups. Emphasis is placed on biomarkers that are statistically different, with the color of the histogram representing the respective group and the length representing the LDA score which reflects the degree of effect of the significantly different species between groups.

We used linear discriminant analysis (LDA) and linear discriminant analysis effect size (LEfSe) methods to investigate the effects of flaxseed ingestion on the cecal microbial community of laying hens (LDA > 3). At three dpi, *Gammaproteobacteria* were identified as the differentially abundant taxa that distinguished the AS group from the others (Figure 5A,F). At seven dpi, *Deltaproteobacteria* were the differentially abundant bacteria in the S group (Figure 5B,G). At 14 dpi, *Rikenellaceae* were the most abundant bacteria in the S group (Figure 5C,H) and at 21 dpi Firmicutes were the primary differential bacteria in the AS group, including *Ruminococcus* and *Lactobacillus* (Figure 5D,I). At 28 dpi, *Lachnospiraceae* and *Erysipelotrichaceae* were the predominant bacteria in the S group (Figure 5E,J).

### 3.4. S. Enteritidis Alters Gene Expression in Ovaries

**Figure 6:**
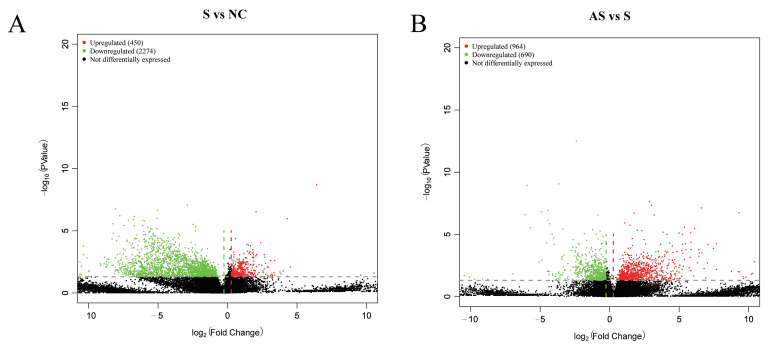
Volcano plot of differential ovarian gene expression at seven dpi. Differentially expressed genes in the S group versus NC group (**A**). Differentially expressed genes in the AS group versus s group (**B**). The horizontal axis represents the fold change (log [B/A]) value of differential gene expression between the different sample groups whereas the vertical axis is the P-value representing the statistical significance of the change in gene expression. Each point on the graph represents a gene where red indicates upregulated genes, green indicates downregulated genes, and black indicates non-differentially expressed genes.

*S.* Enteritidis persisted in the ovaries of the S group and the abundance of *S.* Enteritidis in the AS group was consistently lower than in the S group. Therefore, we further analyzed the effect of flaxseed on gene expression in ovaries of laying hens infected with *S.* Enteritidis by transcriptome sequencing. RNA sequencing was performed on ovarian samples collected at seven dpi and the differentially expressed genes (DEGs) were analyzed using the DESeq method (*q*-value < 0.05, |Fold Change| > 2). A total of 2724 genes were differentially expressed in the S group compared to the NC group, of which 2274 were downregulated and 450 were upregulated (Figure 6A). A total of 1654 genes were differentially expressed in the AS group compared to the S group, of which 964 were upregulated and 690 were downregulated (Figure 6B).

**Figure 7:**
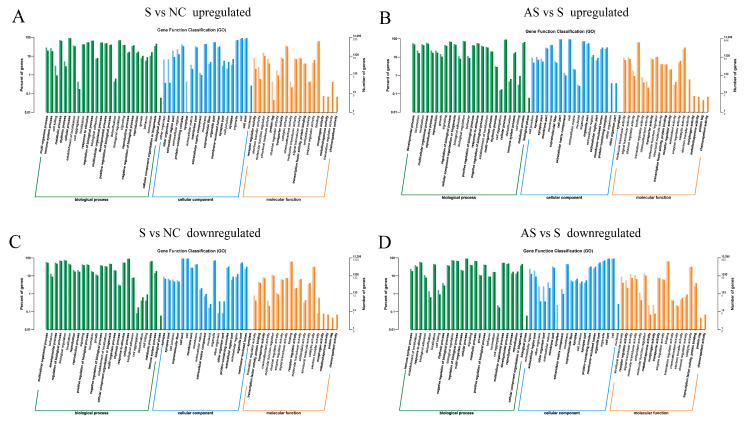
Histogram of the gene ontology (GO) classification of the differential genes. The annotation map of differential genes upregulated (**A**) and downregulated (**B**) in the S group compared to the NC group. Also shown are the differential genes upregulated (**C**) and downregulated (**D**) in the AS group compared to the S group. The horizontal axis shows the functional classification and the vertical axis is the number of genes within that classification (**right**) and their percentage of the total number of genes annotated (**left**). Different colors represent different classifications. Lighter colors on the bars and axes represent differential genes whereas darker colors represent all genes.

Gene ontology (GO) analysis was performed on the DEGs, which were enriched in biological processes, cellular components, and molecular functions. Compared to the NC group, the DEGs upregulated in the S group were primarily enriched in other organism, extracellular regions, and receptor regulator activity (Figure 7A). The DEGs downregulated in the S group were mainly enriched in terms of their developmental processes, biological regulation, and behavior (Figure 7C). Compared to the S group, the DEGs upregulated in the AS group were primarily enriched in functions such as developmental process, localization, and cell junction (Figure 7B). In contrast, the DEGs downregulated in the AS group were primarily enriched in functions such as the extracellular region, other organisms, structural molecule activity, and receptor regulator activity (Figure 7D).

**Figure 8:**
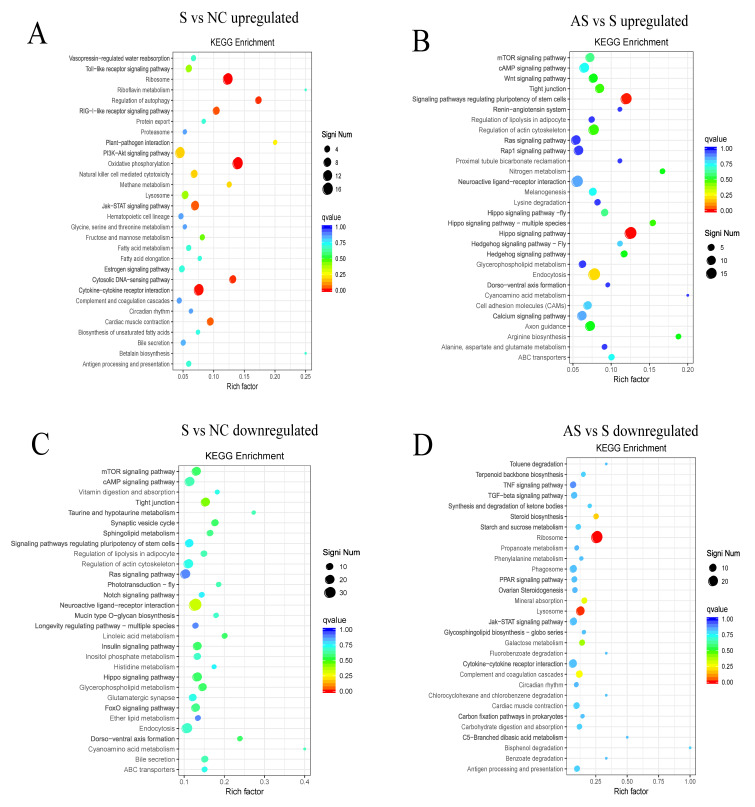
Scatter plot of significantly enriched functions using Kyoto Encyclopedia of Genes and Genomes (KEGG) analysis. (**A**) Upregulated genes in group S versus NC; (**B**) downregulated genes in group S versus NC; (**C**) upregulated genes in group AS versus S; (**D**) downregulated genes in group AS versus S. The vertical axis indicates the function annotation information whereas the horizontal axis indicates the Rich factor. The color of the dot indicates the size of the q-value; the smaller the q-value, the closer the color is to red. The size of the dot indicates the number of differential genes contained under each function.

Kyoto Encyclopedia of Genes and Genomes (KEGG) pathway analysis was also performed on the DEGs. Compared to those in the NC group, the DEGs upregulated in the S group were mainly enriched in terms of oxidative phosphorylation, ribosome, cytokine–cytokine receptor interaction, autophagy regulation, signaling pathways, and myocardial contraction pathways (Figure 8A). Compared to those in the S group, the DEGs upregulated in the AS group were primarily enriched in the Hippo signaling pathway (Figure 8B); the downregulated DEGs were mainly enriched in the ribosomal and lysosomal metabolic pathways (Figure 8D).

**Figure 9:**
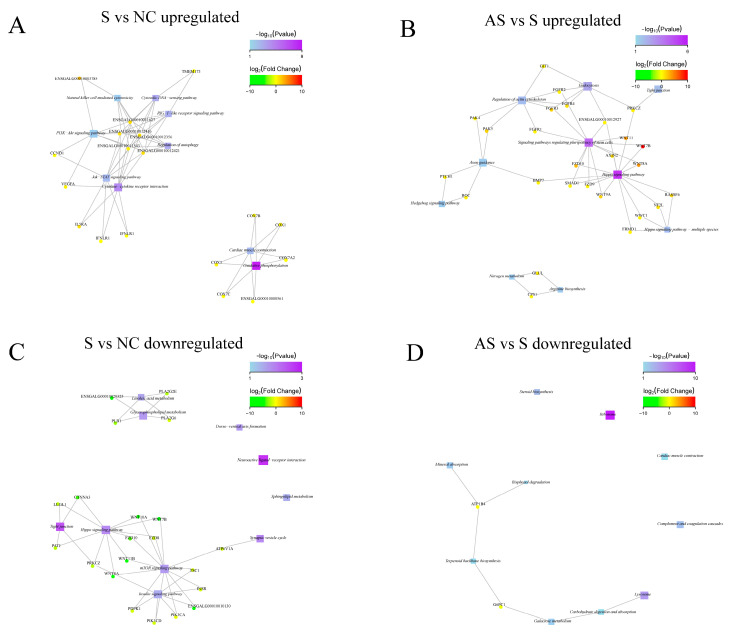
Kyoto Encyclopedia of Genes and Genomes analysis showed the significantly enriched function–gene interactions network graph. (**A**) Upregulated genes in group S versus NC; (**B**) downregulated genes in group S versus NC; (**C**) upregulated genes in group AS versus S; (**D**) downregulated genes in group AS versus S. The square nodes indicate the functional information, the circular nodes indicate the genes, and the edges represent the association between genes and functions. The size of a node is proportional to its degree of connectivity. The color of a round node represents the degree of difference in expression of a gene in that group of samples, with green representing downregulation and red representing upregulation. The shade of the color indicates the degree of difference between up- and downregulation. The color of the square node represents the enrichment of the function, i.e., the P-value; the higher the enrichment, the lower the P-value and the darker the color. The larger the area of the square nodes indicates that more differential genes are involved and thus the more they contribute to the biological phenomenon.

We performed a functional enrichment association analysis to investigate the functions of the DEGs. A significantly enriched function–gene interaction network analysis was also performed. Compared to the NC group, the DEGs upregulated in the S group were associated with host immune response-related functions, such as the cytokine–cytokine receptor interaction, the JAK-STAT signaling pathway, and autophagy regulation (Figure 9A). The *PLB1*, *FZD8*, *FZD10*, and *WNT7B* genes, which were downregulated in the S group, were associated with linoleic acid metabolism, glycerophospholipid metabolism, the mTOR signaling pathway, and the Hippo signaling pathway (Figure 9C). Compared to the S group, the differential genes *FZD10*, *WNT7B*, *WNT8A*, and *WNT11*, which were upregulated in the AS group (Figure 9B), were associated with the Hippo signaling pathway and signaling pathways regulating the pluripotency of stem cells.

### 3.5. Validation of RNA-seq DEGs by Reverse Transcription Quantitative PCR

Among the DEGs, *calr*, *rpl13*, *tagln*, *cd24*, and *rps27a* were selected for validation. The results of the reverse transcription quantitative PCR(RT-qPCR) validation assay were consistent with those of transcriptome sequencing, indicating that the transcriptome sequencing results were plausible (Figure S2).

## 4. Discussion

In recent years, *S.* Enteritidis infections have been prevented and controlled to a certain extent through the establishment of biosecurity barriers, preventive immunization, regular testing, and other measures. However, direct egg contamination and vertical transmission of *S.* Enteritidis are difficult to eliminate and prevent due to the susceptibility of poultry reproductive tracts to infection. In our study, *S.* Enteritidis abundance increased and then decreased in all chicken tissues, reaching a maximum on day seven post-infection. However, *S.* Enteritidis colonization in the flaxseed-fed group was considerably reduced and the clearance cycle in vivo was markedly shortened in this group compared to that in the regular feed group. *S.* Enteritidis was primarily cleared from the laying hens at 21 dpi. In contrast, *S.* Enteritidis was still present in some tissues and organs in the standard feed group at 28 dpi. This result suggests that adding flaxseed may aid in resisting *S.* Enteritidis colonization in laying hens and increase the bacterial clearance rate.

Studies have shown that PUFAs are components of cell membranes and can influence bacterial adhesion by altering the lipid composition in the intestinal wall or bacterial cell wall. The probiotic Lactobacillus plantarum, in combination with flaxseed oil enriched with n-3 PUFAs, exerted an anti-inflammatory effect on the intestinal mucosa of pigs infected with enterotoxin-producing *Escherichia coli* [39]. Continuous supplementation with probiotic cheese enriched with *L. reuteri* CCM 8617RIF and crushed flaxseed had a positive effect on the composition of the intestinal microbiota and mitigated the infectious process induced by pathogenic *Escherichia coli* [40]. Our research shows that the abundance of *S.* Enteritidis was highest in the heart and liver among all tissues and organs, similarly to the results of previous studies [41]. In the present study, HE staining of the ileal tissue was performed. Ileal tissue damage was more severe in the S group than in the AS group, suggesting that adding flaxseed to the feed can alleviate ileal tissue damage caused by *S.* Enteritidis to some extent. The beneficial effects of PUFAs may arise from their anti-inflammatory properties which limit the tissue damage associated with pathogens and inflammatory responses [42].

Intestinal microbiota plays a crucial role in the resistance to exogenous microorganisms through phage deployment, antimicrobial secretion, competitive nutrients, and intestinal barrier function. The cecum is a critical site for functional activity because it has the highest degree of water absorption and carbohydrate fermentation which substantially increases the taxonomic diversity of the microbiota and is vital for health and productive performance of lay hens [43]. Differences in the feed composition, breed, age, and environment can alter the intestinal flora of chickens. Imbalances in the composition of the gut microbiota can lead to a variety of reproductive dysfunctions, such as polycystic ovary syndrome, endometriosis, and ovarian cancer in mammals [44]. Studies have shown that transplantation with fecal microbiota from high laying hens resulted in a significant increase in egg production, suggesting that microbiota may have a positive effect on egg production performance in laying hens and breeders [45]. The gut microbiome of chickens with high egg production performance has more diversity than that of chickens with low egg production performance which may be related to changes in host metabolism and health as well as egg production [46].

Our results suggest that *Salmonella* and flaxseed have long-term effects on the structure of the cecal flora of laying hens. Adding flaxseed to the feed reduced the α-diversity of the intestinal flora of laying hens compared to using the regular feed, consistent with the results of previous studies [47,48]. The α-diversity of the gut flora in the AS group progressively increased at the five different time points, which agrees with previous findings [49]. This alteration may account for the faster recovery from *S.* Enteritidis infection in the AS group.

At the phylum level, Firmicutes and Bacteroidetes were the dominant microbial groups. Firmicutes are primarily anaerobic and the anatomical physiology and feeding habits of chickens can explain the high abundance of Firmicutes [11]. At 3 and 7 dpi, the abundance of Bacteroidetes was higher in the AS group than in the S group. Bacteroidetes strains can produce propionic acid to reduce colonization by *Salmonella* [50], suggesting that flaxseed may have increased the abundance of Bacteroidetes and thus reduced *S.* Enteritidis colonization in laying hens. The relative abundance of Verrucomicrobia was highest in the S group compared to the other three groups at 3, 7, and 21 dpi. *Akkermansia muciniphila*, a member of Verrucomicrobia, is a mucus-degrading bacterium, the abundance of which increases during inflammation [51]. The complementation of *S.* Enteritidis with *Akkermansia muciniphila* resulted in increased inflammation, such as increased histopathological scores and protein and mRNA levels of pro-inflammatory cytokines. Although *Akkermansia muciniphila* is usually considered a beneficial bacterium, it exacerbates *Salmonella* infection by thinning the mucus layer, facilitating pathogen transfer to the upper cortex [52]. This finding indicates that *S.* Enteritidis invasion causes an inflammatory response in the host, accompanied by an increase in the abundance of Verrucomicrobia, which was gradually cleared after 28 dpi, and a decrease in the abundance of Verrucomicrobia.

*Bacteroides* are beneficial intestinal bacteria that strengthen immune function and improve intestinal health [53]. Several *Lactobacillus* strains have been identified as functional probiotics and are implicated in many biological processes, including scavenging pathogenic bacteria, improving intestinal barrier function, anticancer activity, and host metabolic regulation [54]. Flaxseed can enhance the abundance of *Lactobacillus* [55]. At the genus level, the abundance of *Bacteroides* and *Lactobacillus* in the AS group was notably higher than that in the S group. In this study, adding flaxseed to the feed markedly increased the number of beneficial bacteria in the intestinal flora of laying hens, thereby facilitating resistance to invasion and colonization by pathogenic bacteria.

In our study, we found that *S.* Enteritidis persisted in the ovarian tissue of chickens. Therefore, we further investigated the effect of *S.* Enteritidis infection on ovarian gene expression via transcriptome sequencing of the ovarian tissue. Compared with those in the S group, GO analysis showed that the differential genes upregulated in the AS group were primarily enriched in functions such as developmental processes and downregulated in functions such as receptor regulator activity. PUFAs are the main components of cell membranes and play essential roles in muscle cell development [56,57]. Flaxseed oil reduces the mRNA expression of intestinal necrosis signals, such as intestinal toll-like receptor 4 (*TLR4*) and its downstream signal myeloid differentiation factor 88, thereby enhancing intestinal barrier integrity and function [58]. This finding suggests that flaxseed may promote the development of ovarian tissues and may also have a resistance effect on *Salmonella* infection.

Flaxseed fiber alters the cecal microbial ecology, ferments into SCFAs in the cecum, and regulates the transcriptome of colonic endocrine cells, which may contribute to their metabolically favorable phenotype [59]. Cytokines are key triggers of the immune response and inflammation [60] and the JAK-STAT pathway is necessary for the development of T- and B-cells [61]. Autophagy is a crucial defense mechanism for the clearance of intracellular pathogenic organisms and a regulator of the immune response. Following *S.* Enteritidis infection in laying hens, the upregulation of genes interacting with cytokines and the activation of several cytokines in the reproductive organs may lead to a local inflammatory immune response in the ovary.

DNA recognition is an evolutionarily conserved mechanism that initiates a rapid innate immune response against microbial pathogens [62]. Our results show that the upregulated DEGs following *S.* Enteritidis infection were primarily enriched in metabolic pathways related to host immunity, such as the JAK-STAT signaling pathway, whereas the downregulated genes were mainly enriched in fatty acid metabolism. Linoleic acid exhibits anti-inflammatory activity [63]. Glycerophospholipid metabolism is one of the most tightly regulated metabolic processes throughout a 24 h cycle in terms of total lipid content, enzyme expression, and activity in the nervous system and individual cells, with multiple physiological functions and disease implications [64]. The mTOR signaling pathway regulates immune function and T-cell differentiation by integrating various microenvironmental signals [65]. The Hippo pathway is a crucial tissue growth regulator, is evolutionarily conserved, and regulates many biological processes, including cell growth, fate determination, organ size control, and regeneration [66]. *S.* Enteritidis infection in laying hens resulted in the significant downregulation of *plb1*, *pla2g2e*, and *pla2g6* which affected host lipid metabolism. This finding suggests that *Salmonella* infection disrupts the cellular mechanisms involved in nutrient synthesis, uptake, and metabolism, similar to previous findings [67]. In addition, the invasion of *S.* Enteritidis affects tissue growth and host development.

Compared to those in the S group, the upregulated DEGs in the AS group were mainly enriched in the Hippo signaling pathway which regulates the pluripotency of stem cells. The Hippo signaling pathway also regulates ovarian physiology, fertility, and pathology [68]. Our results suggest that adding flaxseed to the feed of laying hens infected with *S.* Enteritidis may promote ovarian growth and development. *S.* Enteritidis infection in laying hens resulted in the downregulation of some DEGs, such as *wnt7a*, *wnt8a*, and *fzd10*, which are primarily involved in the mTOR and Hippo signaling pathways. However, the expression of these genes was upregulated in the group treated with flaxseed, suggesting that adding flaxseed to the feed may alleviate the adverse effects of *S.* Enteritidis on laying hens.

Incorporating flaxseed into the feed of laying hens has demonstrated various advantages. Based on previous studies, the recommended level of flaxseed in feed for laying hens is (15% [750 mg/kg]) [22,23]. It not only reduces the colonization of *S.* Enteritidis and minimizes damage to the ileal tissue but also promotes the abundance of beneficial intestinal flora. Moreover, flaxseed positively impacts ovarian gene expression. The findings of this study have substantial implications for large-scale farms, aiding them in more effectively preventing and treating *S.* Enteritidis infections. Furthermore, the utilization of flaxseed can enhance the production performance of laying hens, which is of utmost importance in both public health and agriculture sectors.

## Figures and Tables

**Table 1:** Primers used for quantitative real-time PCR validation.

Genes		Primer Sequence (5′–3′)	Tm (°C)	Product Length (bp)
*GAPDH*	F	GCCCAGAACATCATCCCA	56.4	137
	R	CGGCAGGTCAGGTCAACA	57.7	
*CALR*	F	GGAAGTTCTACGGCGATGCT	59.5	150
	R	CGATGTTCTGCTCGTGTTTGA	59.5	
*CD24*	F	GATCCCAATGGAACAAGTC	51.7	128
	R	CTCGTGGTGGAGTGAAGG	53.3	
*RPS27A*	F	GGAAGACCATCACCCTCG	55.1	195
	R	CACGCAGTCTCAGCACAA	53.3	
*TAGLN*	F	CCAGACCGTTGACCTCTTTGA	60.0	155
	R	AGAACTCCCGCTTGTGCTCC	60.0	
*RPL13*	F	GCCCGACTGTCAGATACCA	56.0	102
	R	CAATCGTCCGAGCAAACC	56.7	

## Data Availability

The 16S rRNA amplicon sequencing data obtained in this study have been deposited in the National Center for Biotechnology Information (NCBI) database under the BioProject accession no. PRJNA956852. The raw data for the transcriptome analysis have also been submitted to NCBI under BioProject accession no. PRJNA957106.

## Supplementary Materials

The following supporting information can be downloaded at: https://www.mdpi.com/article/10.3390/biom13091353/s1, Figure S1: Analysis of the alpha diversity of flaxseed on the microbiota of the cecum contents of chickens infected with *S.* Enteritidis; Figure S2: Real-time polymerase chain reaction (PCR) validation for the functional genes. Table S1: Wilcoxon rank-sum test for changes in microbial abundance of cecal contents at the phylum and genus level.
