# Supplementary figures and images for: Flaxseed Supplementation in Chicken Feed Accelerates Salmonella enterica subsp. enterica Serovar Enteritidis Clearance, Modulates Cecum Microbiota, and Influences Ovarian Gene Expression in Laying Hens
Source: Biomolecules. 2023 Sep 6;13(9):1353. doi: 10.3390/biom13091353 (PMC10526464; doi:10.3390/biom13091353)

A

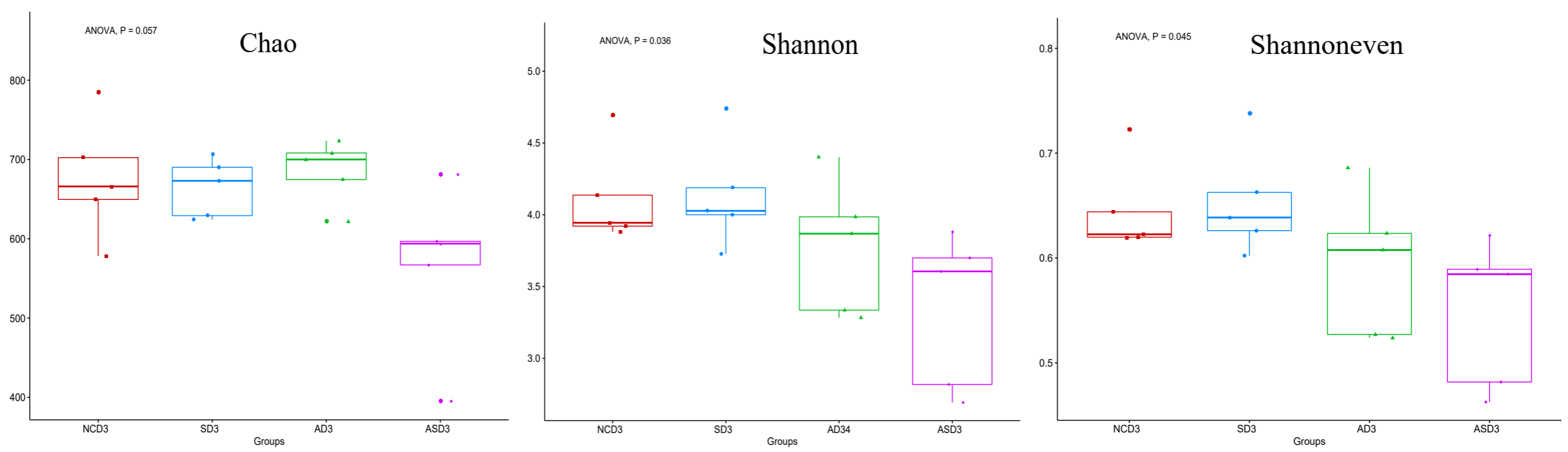

B

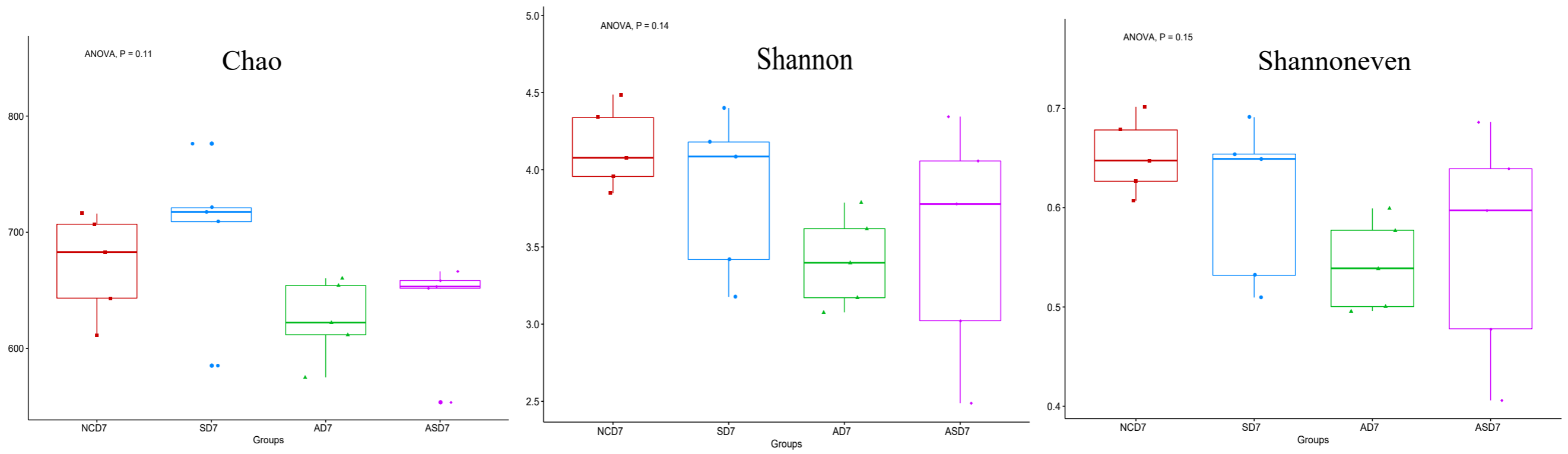

C

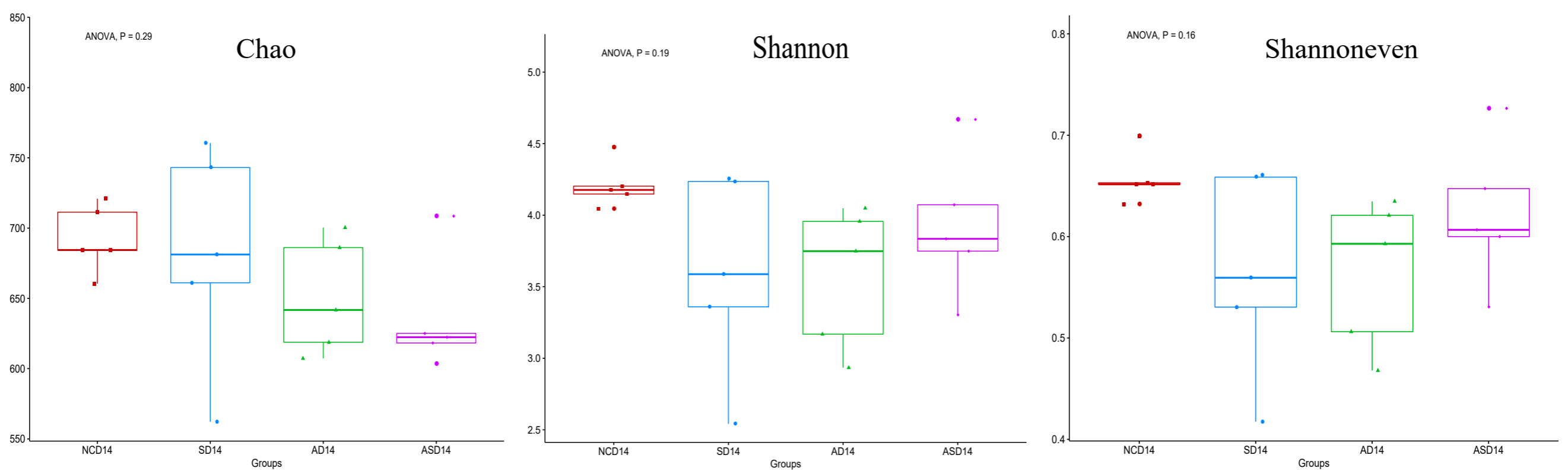

D

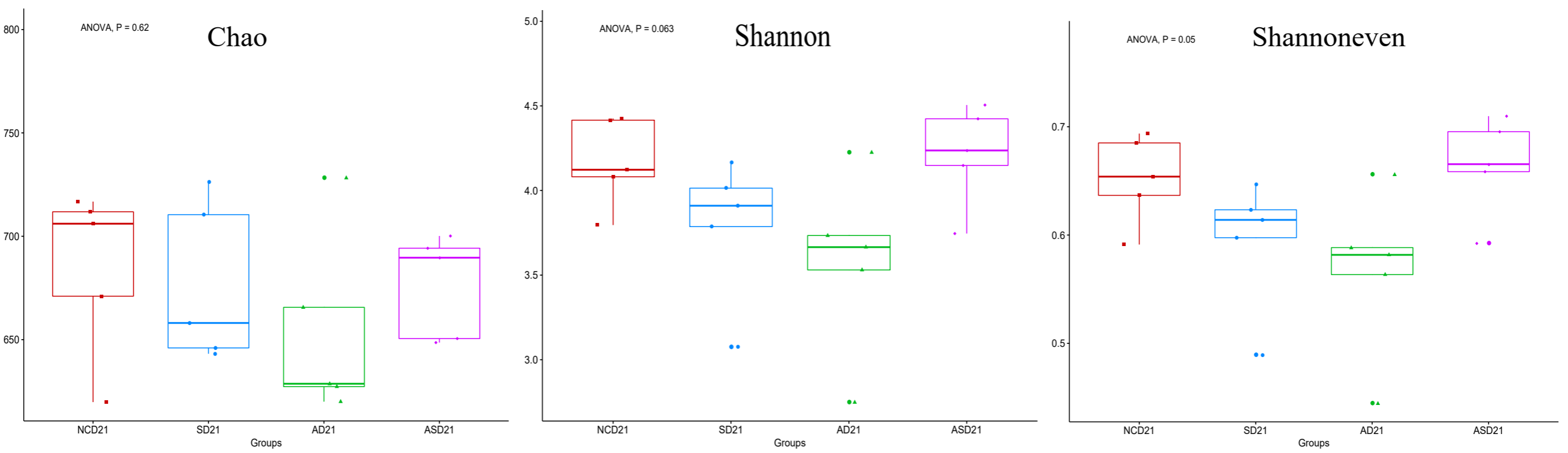

E

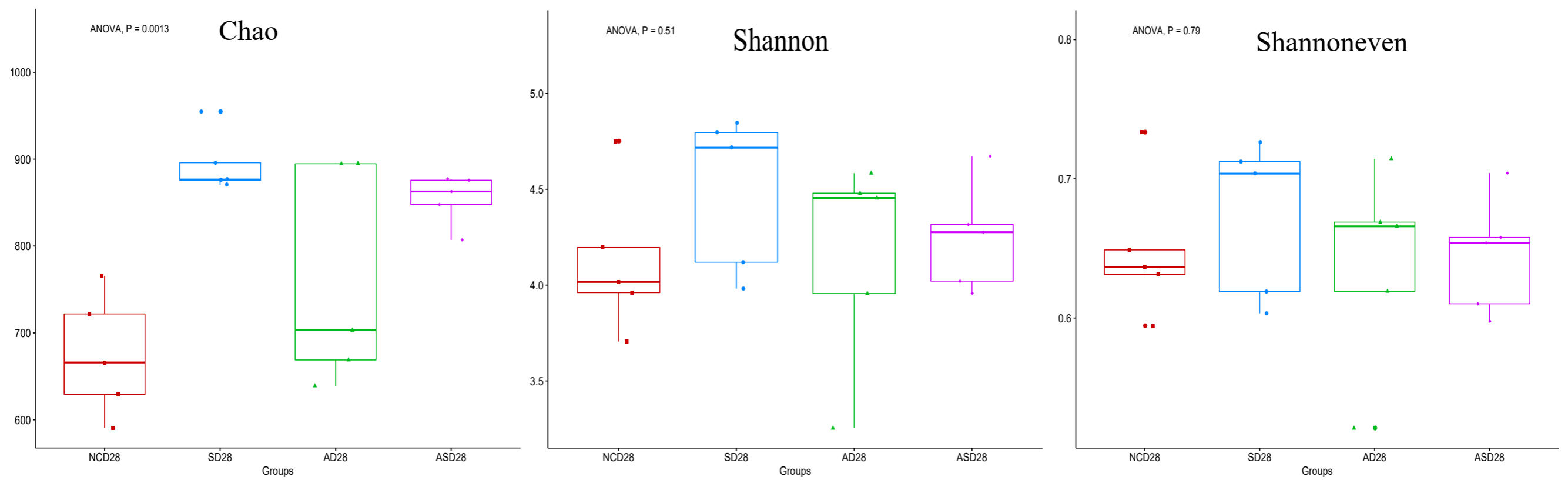

Supplement: Supplementary file 1 [file biomolecules-13-01353-s001.zip › biomolecules-2514822-Supplementary Material/FigureS1.pdf]

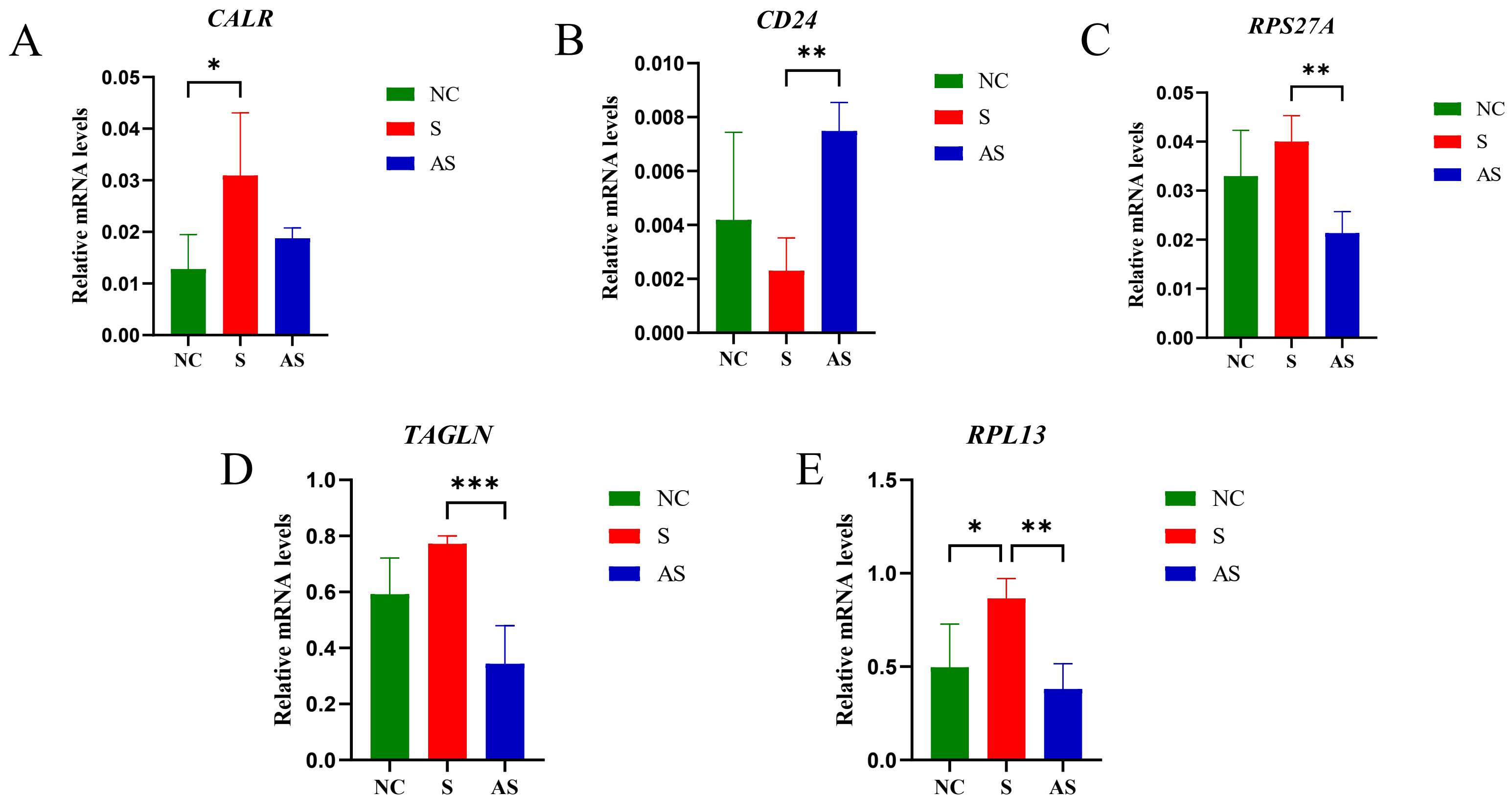

Supplement: Supplementary file 1 [file biomolecules-13-01353-s001.zip › biomolecules-2514822-Supplementary Material/FigureS2.pdf]
